# Physical Activity as an Intervention for Frailty Syndrome: A Narrative Review

**DOI:** 10.7759/cureus.100293

**Published:** 2025-12-28

**Authors:** Reuben Oza

**Affiliations:** 1 Geriatrics, University of Birmingham, Birmingham, GBR

**Keywords:** aging, exercise therapy, frailty syndrome, geriatrics, physical activity

## Abstract

Frailty is a geriatric syndrome characterised by a decline in functional reserves as the body ages, resulting in increased disability, comorbidity, and mortality. With trends towards ageing populations, frailty syndrome becomes more clinically relevant, highlighting the importance of appropriately preventing and managing the characteristics of frailty syndrome.

Risk factor modification is recommended to delay or prevent the onset of frailty, including physical activity alongside other modifiable behaviours such as diet. Ageing is associated with chronic low-grade inflammation, resulting in reduced muscle protein synthesis and increased resistance to insulin, which both contribute to sarcopenia. Sarcopenia underpins key characteristics of frailty, including weakness and slow speed. Physical activity stimulates anabolic pathways and improves insulin resistance, reducing sarcopenia. Moreover, aerobic exercise is responsible for increasing the VO_2_ peak, whilst resistance exercise improves muscle strength, both of which are known to decrease in frail elders.

This narrative review primarily explored the effectiveness of physical activity in reducing the risk of the onset of frailty syndrome through a narrative review of the relevant literature concerning this subject. A secondary focus of this narrative review is to compare the success of alternative interventions for preventing frailty, relative to physical activity.

Physical activity interventions have been shown to improve components of frailty scoring and selected biological markers of frailty, with evidence suggesting physical activity is an effective single-domain intervention for frailty; however, multidomain approaches may result in a greater overall improvement in frailty prevention. Further research is required to identify the types of exercise that modify specific aspects of Fried et al.’s frailty criteria (FFC), as well as what interventions can be used alongside physical activity, to holistically treat all characteristics of frailty syndrome.

## Introduction and background

Defining frailty

Frailty syndrome is a geriatric syndrome displaying a distinct phenotype. Fried and colleagues described a characteristic phenotype for frailty, in which patients can be classified according to the presence of five criteria [1]. Fried et al.’s frailty criteria (FFC) include weakness, low physical activity, involuntary weight loss, slow gait speed, and exhaustion. A patient who presents with three or more of the above characteristics can be classified as ‘frail’, whilst those with one or two characteristics are classified as ‘pre-frail’. Alternatively, Rockwood and colleagues designed the ‘frailty index’ [2], which uses multiple domains including the incidences of deficits such as disabilities, geriatric syndromes (i.e., delirium), diseases, and various psychosocial factors related to that patient. These deficits are used in the formula, ‘n. of deficits present/n. of possible deficits’, to give the frailty index. In contrast to FFC, which divides patients into categories, the frailty index produces a continuous score estimating the degree of deficit accumulation. With no one universally accepted method for diagnosis and/or grading of frailty, jointly considering these models that address both the physical expression of frailty and its relation to age-related processes is important when evaluating preventative interventions. Using these definitions of frailty, it can be inferred that frailty denotes a syndrome characterised by a decline in functional reserves as the body ages, which increases the probability of disability, comorbidity, and mortality [3].

Difference between frailty and chronological age 

As opposed to measuring chronological age, frailty is an indicator of biological ageing and ageing-associated processes, such as those included in FFC and the frailty index, which typically decline as age increases [4]. Secondary analysis of the English Longitudinal Study of Ageing identified an increase in the prevalence of frailty (defined using FFC) with increasing biological age [5]. Frailty was present in 65% of participants aged 90 years or older, compared to being present in just 6.5% of participants aged between 60 and 69 years. Therefore, as the UK population continues towards an increasingly ageing demographic [6], there is an increased need to prevent and manage the characteristics of frailty syndrome. National Institute for Health and Care Excellence (NICE) clinical guidance (NG16) advises risk factor modification to prevent frailty, including recommending physical activity, alongside other contributing factors [7]. NG16 does not provide comparative evidence on the relative effectiveness of individual preventative interventions, highlighting the role of this review in evaluating intervention-specific evidence.

How physical activity prevents frailty 

There are numerous mechanisms by which physical activity is thought to act as an intervention for frailty syndrome. Ageing is associated with chronic low-grade inflammation, resulting in reduced muscle protein synthesis and increased resistance to insulin [8], which both contribute to sarcopenia. Sarcopenia underpins key characteristics of frailty within FFC, including weakness and slow gait speed [9]. Physical activity mitigates sarcopenia by increasing muscle protein synthesis via stimulating anabolism. Moreover, aerobic exercise is responsible for increasing the VO_2_ peak, whilst resistance exercise improves muscle strength, both of which are known to decrease in frail elders [10]. Physical activity primarily improves weakness and slow gait speed, key components of FFC, providing a rationale for frailty interventions centred around physical activity. This review primarily explores the effectiveness of physical activity in reducing the risk of the onset of frailty syndrome through a narrative review of the relevant literature concerning this subject. A secondary focus is to compare the success of alternative interventions for preventing frailty, relative to physical activity.

This narrative review was completed as an academic project within the University of Birmingham MBChB Programme, during the 'Personal Interest Project' module in 2020/2021.

## Review

Methods

This narrative review synthesised evidence on the role of physical activity in the prevention and management of frailty in older adults. Literature was identified through targeted searches of widely used biomedical literature databases, including MEDLINE and Embase, followed by manual screening of reference lists from relevant studies identified. Searches were conducted during the period in which the review was undertaken (2020-2021) and focussed on studies involving frailty, physical activity or exercise interventions, and ageing populations. Study selection was influenced by relevance to the aim of the review rather than predefined inclusion or exclusion criteria. No formal meta-analysis or risk-of-bias assessment was undertaken, and findings were narratively synthesised, with a focus on study design, outcome measures, and consistency of reported effects across studies.

Reviewing the effectiveness of physical activity as an intervention for frailty 

Randomised Controlled Trial One 

The lifestyle interventions and independence for elders pilot (LIFE-P) randomised controlled trial (RCT) assessed the success of physical activity in promoting healthy ageing in 424 sedentary individuals between the ages of 70 and 89 years [11]. The community-dwelling participants were randomly assigned to an intervention or active control group. Within the intervention arm, participants carried out an endurance exercise, typically walking, for a minimum of 150 minutes/week, which was then followed by strength, balance, and aerobic training, over a period of 12 months. In contrast, the control arm attended educational meetings regarding healthy behaviours in old age but did not follow a physical activity routine. At baseline, FFC was recorded for each participant, with no statistical differences found, and this was repeated every six months as a measure of the success of the intervention. A subsequent exploratory analysis found that after 12 months, a significant (p = 0.01) reduction in the prevalence of frailty was observed within the intervention arm compared to the control arm [12]. Frailty prevalence in the physical activity group was 10.0% compared to 19.1% in the control group. Given that this cohort was predominantly ‘pre-frail’ at baseline, these findings will be evaluated as preventing or slowing frailty progression, rather than evaluating reversal of established frailty. With a reduction in frequency and extent of frailty, this study supports the effectiveness of physical activity in improving selected components of FFC, particularly sedentary behaviour.

The use of FFC as a measure of frailty was particularly useful. Within current practice, there is a lack of a clear definition of frailty, which creates difficulties in accurately identifying the best possible intervention, yet recent literature proposes that interventions that target FFC may be effective in preventing the progression of, or reversing, frailty [13]. This highlights the usefulness of this source in assessing the impact of physical activity on frailty syndrome, contextualising the effectiveness within a globally understood model whilst utilising the intervention in a manner shown to be most successful. Furthermore, allocation bias and attrition bias were minimised via randomly assigning participants to either arm, with randomisation stratified by factors such as gender, and by the high retention rate of participants, respectively; these were further strengths of this RCT. The sample size was much larger, relative to other investigations included within this review, which allowed for a greater reduction in the margin of error, thus ensuring more accurate results and identification of anomalies. However, the exclusion criteria resulted in those with cognitive disorders being omitted from the study. A 2005 study determined that approximately 20% of their sample of 15,051 individuals aged over 75 had cognitive impairment, which suggests that the results of this study display a poor generalisability across an elderly population, therefore lowering the sampling validity of these results as they may not be applicable to a large portion of elderly individuals [14].

Notably, further analyses indicated that physical activity did not improve all aspects of FFC. After 12 months, although a significant decrease in the mean number of FFC was observed for the intervention arm, only the sedentary behaviour of participants was significantly improved as a result of physical activity, whereas other characteristics of FFC were not improved [12]. Therefore, whilst this physical activity can reduce sedentary behaviour, which may lead to a reduction in frailty, this study demonstrates that the physical activity intervention used will not be suitable for all pre-frail or frail individuals, as sedentary behaviour may not be a characteristic of the frailty syndrome that they exhibit. Furthermore, modifying one risk factor does not dispel the harmful effects that characteristics such as unintentional weight loss can have for frail adults. This is not to say that physical activity is an ineffective intervention for frailty, but rather the intervention needs to be modified to improve more aspects of FFC, to be conclusively determined as a holistic intervention for frailty syndrome.

Overall, it can be concluded that despite the aforementioned limitations of physical activity as an intervention, this study provides important evidence that physical activity can improve selected components of FFC, namely, sedentary behaviour, which may contribute to reduced prevalence of frailty amongst at-risk elderly. It is important to note the limited change observed in other components of FFC. The findings of this study are supported by a systematic review of 47 RCTs investigating the effectiveness of physical activity interventions, which found that structured exercise training reduces frailty and is a suitable intervention [15]. Perhaps different types of exercise or intensity or alternative interventions altogether could be combined with this RCT methodology to produce a more efficient intervention for frailty syndrome. This is a particularly important question raised by this evaluation and will be investigated towards the latter part of this review.

Randomised Controlled Trial Two

Conversely, a 2003 RCT used immune parameters, rather than FFC, as an outcome measure to assess whether a 32-week, moderate physical activity intervention for 190 frail elderly would be successful in reversing frailty [16]. Over eight months, the intervention group aimed to complete four sessions of resistance and endurance exercise, five days/week, whereas the control group received no such structured exercise training. At baseline, blood samples were taken to identify the level of immunological markers such as lymphocyte subpopulations, activation markers, and T-cell proliferation. This process was repeated after eight weeks, and at the end of the intervention, to identify the final effects of the intervention. After 32 weeks, no significant difference was observed in lymphocyte subpopulations, activation markers, or T-cell proliferation at any point in the intervention, indicating that physical activity is not effective in combatting the immunosenescence associated with frailty [17]. As immune parameters are not established markers of frailty reversal, these null findings do not negate the observed improvements in FFC described in other studies; however, they do limit our ability to make conclusions on the effect of physical activity on immunosenescence.

Allocation bias was negated via randomly assigning participants to each arm, whilst variables such as diet and circadian rhythm were controlled for all participants. This is particularly important to note because vitamin E-rich foods have been shown to reduce immunosenescence [18], and therefore, each participant received similar meals, whilst blood tests were carried out at the same time of day for each participant to prevent the regular circadian fluctuations in immune cell levels from interfering with results. Controlling these variables facilitated a more accurate insight into the sole effect of physical activity on frailty syndrome. Another strength of this study was that the incidence and severity of upper respiratory tract infections within each arm were monitored, which served as a secondary measure of differences in immunological functioning between the two groups. After the 32-week exercise programme, a significant difference in the incidence rates before and after the intervention was not observed, demonstrating that physical activity had negligible effects on the frail nursing home residents’ immune parameters.

Despite the aforementioned strengths, this RCT did have limitations that challenge the validity of its conclusion. It is possible that the physical activity intervention was not performed for a sufficient length to mirror the results of other studies, such as a two-year moderate physical activity intervention for elderly women, which caused increased production of IL-2, a key regulator of the immune response [19]. Additionally, the lack of long-term follow-up is potentially responsible for no significant change being observed and may have prevented observations from being seen in changes to compliance and patient motivation, which are both critical components of an effective intervention. van den Biggelaar et al. suggest that the onset of frailty in the elderly is due to a faulty immune system [20], highlighting that the significance of the inability of the physical activity intervention in producing a beneficial response to immune functioning is not to be overlooked when considering the effectiveness of this intervention. Perhaps a more interesting perspective when determining the success of physical activity is that physical activity is an intervention that needs to be in place before the onset of frailty, as studies have shown physical activity to improve immune function in non-frail elderly [21]. Therefore, an enlightening area for future research would be the impact of physical activity on immunological parameters of pre-frail or healthy adults, which would provide a better insight into whether physical activity could act to prevent the immunosenescence associated with frailty syndrome if carried out before frailty syndrome presents.

Research has shown that frailty is associated with a greater incidence of infections, which are more difficult to recover from, as a result of deterioration of cell-mediated and humoral immunity as the body ages [17], with lower lymphocyte proliferation common amongst frail patients [22]. For physical activity to be viewed as an effective intervention with regard to this study, significant changes in the immunological markers would need to be seen as opposed to a change in FFC. Therefore, this study provides a well-rounded insight into the effects of physical activity on frailty syndrome, assessing a different aspect of measuring frailty. This RCT suggests physical activity alone may be insufficient to modify the immunosenescence in already frail patients.

Randomised Controlled Trial Three

Incorporating both outcome measures of the above two RCTs, FFC and biological markers of frailty, a 2016 RCT aimed to conclude whether a multicomponent exercise programme (MEP) for 100 frail elderly participants would be successful in improving frailty and functionality [23]. Results after the 24-week intervention concluded that whilst no one from the control group reversed frailty after the six-month programme, 31.4% of the intervention arm reversed frailty, as well as showing a significant improvement in levels of two important frailty biomarkers, D-dimer and plasma protein carbonyls, which was not seen in the control group. This study is particularly relevant towards answering this review question as it provides a holistic insight into the effect of physical activity as an intervention, demonstrating that a structured, multicomponent physical activity programme can both reverse frailty according to the FFC and improve biological markers in a subset of frail older patients.

An important strength of this study was the model of intervention provided. The MEP involved a variety of exercise types including aerobic, strength, flexibility, and proprioception training. Incorporating many different types of exercise into the programme can widen the knowledge base regarding which exercise type is most effective in combatting frailty [24]. It can be argued, however, that despite successful results, this study fails to identify the most effective physical activity intervention, as it is difficult to assess the specific positive and negative impacts of the different exercise types on frailty and overall health and well-being. Due to the intervention combining multiple exercise modalities, the relative contribution of individual components cannot be isolated, limiting inference regarding which elements drive benefit. A particular point of interest with this study is that despite a compliance of less than 50% with the exercise programme, results supporting the success of physical activity as an intervention for frailty were obtained, suggesting that a less intense, more achievable physical activity intervention could be as successful an intervention. This reduced intensity may allow for an intervention that is more feasible for a larger population of frail elders, possibly including those who were excluded from this RCT due to being deemed too ill to perform the prescribed exercise. This is an area for future research that, if validated, has revolutionary implications for establishing the most effective physical activity intervention for frailty.

A limitation to note is the lack of long-term follow-up. The intervention only lasted six months, which prevented long-term consequences of physical activity from being established within the results. Potentially, the lack of change observed in the incidence of geriatric syndromes or falls (common components of frailty) was due to the inadequate follow-up period. The biological markers measured were the levels of D-dimer and plasma protein carbonyls, providing an insight into the amount of clotting and plasma protein oxidation occurring, respectively, which are both known to increase in frail patients [25,26]. The reduction observed in the level of biological markers, coupled with the reversal of frailty, demonstrates that structured, multicomponent physical activity programmes act to not only improve frailty according to Fried et al.’s frailty phenotype but also improve the physiological characteristics associated with frailty. Overall, this study demonstrates the effectiveness of physical activity as an intervention, supporting the conclusion of a systematic review of 20 physical activity intervention RCTs, which determined that multicomponent exercise interventions appear to be among the most effective approaches for improving physical frailty [27]. The validity of these findings is strengthened via the randomisation of participants to either arm, in order to prevent allocation bias, as well as by controlling diet via providing similar meals, to reduce the effect of a confounding variable [28].

Reviewing the effectiveness of alternative interventions for frailty relative to physical activity

An RCT involving pre-frail and frail elderly aimed to determine the success of nutritional, physical, and cognitive interventions in reducing frailty progression, using FFC as an outcome measure [29]. Two hundred forty-six participants were randomised between five different six-month interventions: physical, cognitive, nutritional, combination (all three aforementioned interventions combined), and control intervention groups. The physical intervention involved various exercise types and the nutritional intervention focussed on increasing micronutrient and protein-calorie intake, whilst the cognitive intervention was centred around improving problem-solving, reasoning, and information-processing skills. After 12 months, all interventions resulted in a significant reduction in the frailty score, with the largest improvement observed by the combination group, followed by the physical, nutritional, and cognition intervention groups, respectively. However, the study primarily reported directional changes and relative ranking between intervention arms, without formal between-group effect estimates, limiting precise quantitative comparison between the domains.

A low drop-out rate meant attrition bias was minimised, whilst the exclusion criteria resulted in only 5% of participants having been in a hospital within the 12 months prior to the start of their intervention, which eliminated recovery from illness/injury, acting to improve their frailty score. This ensured that the results seen were due to the interventions alone. In contrast to the previous RCTs evaluated, this study consisted of a majority of pre-frail patients, which demonstrates the effectiveness of each intervention in preventing frailty progression, as opposed to reversing established frailty. Healthcare aims to target prevention rather than treatment where possible [30], as not only does it reduce pressures on the NHS, but it also increases healthy life expectancy and quality of life. Therefore, by demonstrating the effectiveness of each intervention in reducing FFC before frailty onset, this study is particularly relevant to current clinical practice. However, there are limitations of this RCT to note. The high compliance rates seen throughout all intervention groups are often hard to obtain for interventions aimed at the elderly [31], and the study was conducted in Singapore, where the participants selected had relatively good physical and cognitive ability before the intervention. These sample characteristics may not be displayed in all areas of clinical practice, therefore negatively impacting the generalisability of this study, lowering the external validity of the results obtained.

Nonetheless, this study acts as a suitable comparison of alternative interventions for frailty. Results display that in comparison to nutrition or cognitive interventions, physical activity is more effective for reducing frailty progression; however, the combination group reduced their frailty score the most, signifying that physical activity alone is not the most effective solution for reducing frailty progression. This notion is reinforced by the fact that different interventions led to different improvements in FFC. For example, whilst a physical intervention improved gait speed, a nutritional intervention better improved sedentary behaviour, illustrating that whilst physical activity may have the largest effect on FFC as a single-domain intervention, an intervention which aims to improve physical activity, nutrition, and cognition can better target different aspects of FFC, helping to holistically treat the frail patient, eliminating all areas of ill-health concern. A 1994 RCT determined that whilst physical activity alone can counteract physical frailty, nutrition interventions require simultaneous exercise to reduce physical frailty [32], highlighting that physical activity may be the most impactful single-domain intervention. However, overall multidomain approaches combining physical, nutritional, and cognitive components are more effective overall for reducing frailty progression.

Few studies directly compare the relative success of physical activity with other interventions for frailty; however, those investigating more than one intervention-style, for example, a systematic review that analysed 21 RCTs describing interventions centred around physical exercise, nutrition, and cognitive training, observe that each of these intervention types shows signs of reducing frailty indicators [33]. This, therefore, raises the premise of focussing future investigation on identifying which intervention to use, and when, to target specific aspects of frailty syndrome where necessary. This area of study is arguably a more important point of focus for future research as opposed to comparing which is more effective, as there is significant evidence base supporting the effectiveness of combining interventions to reduce frailty.

Limitations

This review is subject to limitations. As a narrative review, a systematic or exhaustive summary of literature has not been undertaken, and no formal risk-of-bias assessment was performed. Several cited studies were published over 10 years ago, and whilst these studies are foundational to frailty research, their date of publication may limit direct generalisability to some modern clinical contexts. In addition, heterogeneity in frailty definitions and outcome measures across incorporated studies limits direct comparison of intervention effectiveness and precise quantitative synthesis.

## Conclusions

This review synthesises evidence supporting physical activity as an effective intervention for frailty syndrome through improving select components of FFC, which is supported by a wide range of evidence-based literature. Available evidence suggests physical activity is an impactful single-domain intervention for reducing frailty progression; however, multidomain approaches demonstrate greater overall improvements in frailty in comparison to any single-domain approach. Despite presenting no conclusive evidence in preventing the immunosenescence associated with frailty, physical activity interventions repeatedly demonstrated an ability to improve FFC and also managed to improve crucial biological markers of frailty in selected studies. However, this review has raised further questions for future investigations, which, if answered, will strengthen the evidence base for the effectiveness of physical activity. These include exploring the types of exercise that modify specific aspects of FFC, as well as what interventions can be used alongside physical activity, to holistically treat all characteristics of frailty syndrome. Frailty is a condition that impacts health outcomes in a similar manner to comorbidities, as well as having the power to lead to a rapid deterioration in quality of life. This highlights the importance of not only understanding the implications of frailty but also striving towards preventing and reversing such a condition where possible.
